# Supplementary figures and images for: XOL-1 regulates developmental timing by modulating the H3K9 landscape in C. elegans early embryos
Source: PLoS Genet. 2024 Aug 15;20(8):e1011238. doi: 10.1371/journal.pgen.1011238 (PMC11349215; doi:10.1371/journal.pgen.1011238)

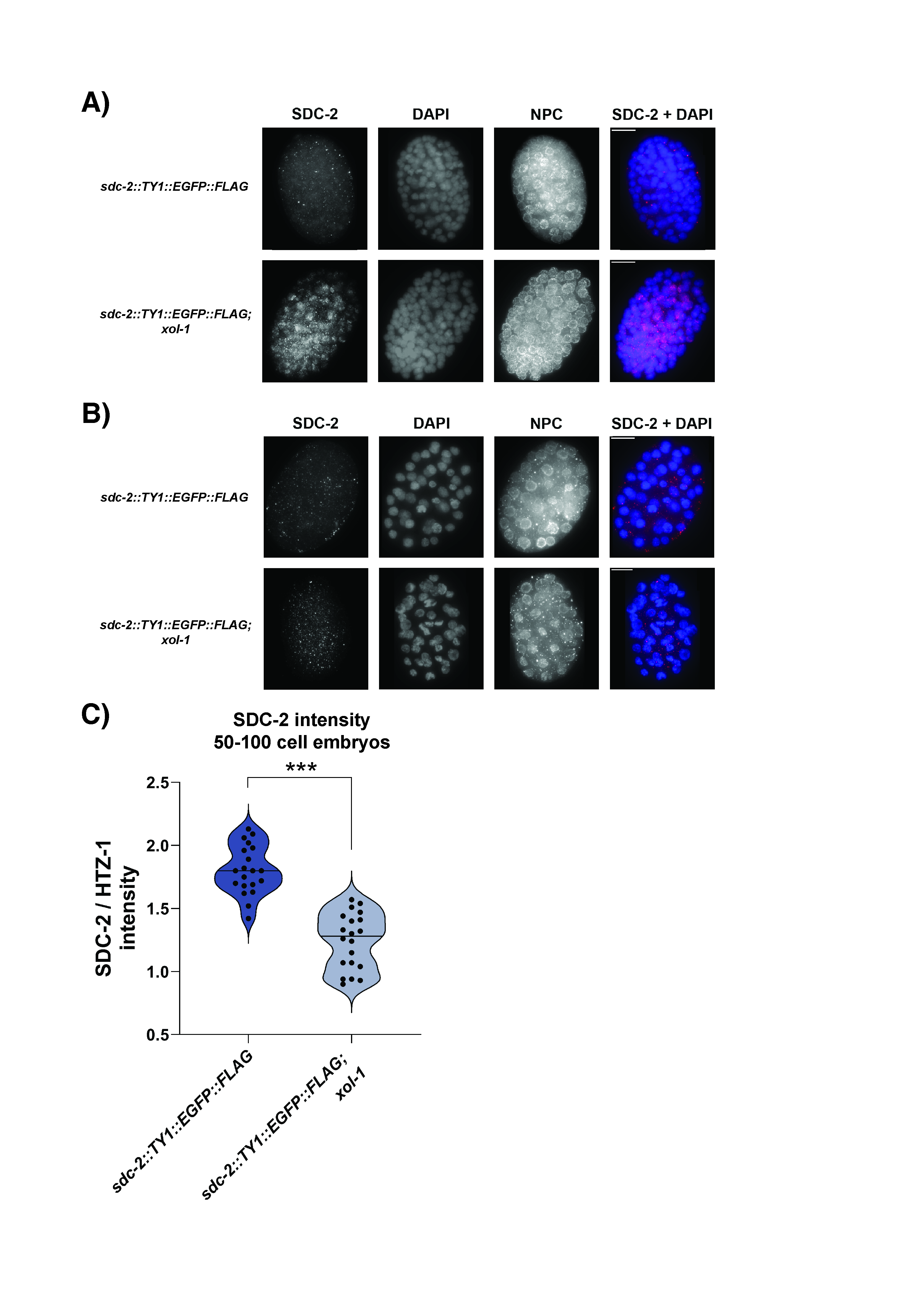

Supplement: S2 Fig — (A-B) Representative images of SDC-2 loading assay quantified (A) Fig 3F, 50–100 cell embryos and (B) Fig 3G, 20–50 cell embryos using sdc-2::TY1::CeGFP::FLAG and sdc-2::TY1::CeGFP::FLAG; xol-1 embryos. Staining against the nuclear pore complex (NPC) was used as an internal control. (C) SDC-2/HTZ-1 intensity quantification of staged N2 and xol-1 embryos at 50–100 cells. P-value determined by Welch’s t-test with two-tailed distribution and unequal variance. p = 2.5 x 10^−10^. 20 embryos were quantified in total for each genotype. Asterisks indicate level of statistical significance (⁎ p<0.05; ⁎⁎ p<0.005; ⁎⁎⁎ p<0.001, n.s not significant). (TIF) [file pgen.1011238.s002.tif]
