# Supplementary material for: XOL-1 regulates developmental timing by modulating the H3K9 landscape in C. elegans early embryos
Source: PLoS Genet. 2024 Aug 15;20(8):e1011238. doi: 10.1371/journal.pgen.1011238 (PMC11349215; doi:10.1371/journal.pgen.1011238)
Supplement: Attachment — Submitted filename: Reviewer responses_GC_EJ2.docx [file pgen.1011238.s005.docx]

Dear Editors and Reviewers,

Thank you for your insightful comments and valuable feedback. We believe we have addressed all the concerns raised by the reviewers in our revised manuscript. We have included a file with track changes to make the changes in the revised text clearer to the reviewers.

In addition, we have generated a private secure token to allow the reviewers to access GSE262626, which contains our RNA-seq raw data. The code to access the GEO submission is gxajegkuthchlyl. We will make this submission public as soon as the paper is accepted for publication.

Reviewer #1: This is a well written paper with extensive introduction that provides background to the system. The main idea is that xol-1 which turns on the male development pathway has a role in delaying hermaphrodite development. This delay is in part due to met2 overexpression, thus may be linked to premature formation of heterochromatin. Although not very mechanistic, this is a nice story and observation that will be of interest to sex determination and embryonic development fields. A few minor comments for revision:

- “known to” repetition in lines 114-116. Could remove one or both.

We have removed the second instance of “known to”.

- in Figure 1C, what is plotted on the y axis? Legend says normalized transcript counts, but it is not clear normalized to what? Thus, one cannot assess what a number on the y axis means.

The y axis plots the dcpm (depth of coverage per base per million reads) for individual transcripts reported in the time-resolved embryogenesis transcriptome dataset generated by Boeck et al. (2016). The dcpm represents expression of transcripts normalized to the read depth of the sample and length of the transcript. We have updated the y-axis on Figure 1C to clarify units of measurement, as well as the y-axis on Figure 5B and Supplementary Figure 4B which plot similar dcpm transcript profiles.

- grammar in line 377 “the disruption male biased pathways”

We have corrected the grammatical error.

- In histone modification change plots of Figure 6 and elsewhere, the authors should consider using violin plots or plots using individual data points to convey the variability of the data better than the bar plots. The bar plots do not provide insight into the spread of the data. In time to develop plots of Figure 7, it will be better to use individual data points, as few embryos are scored.

We have changed all the bar plots with fewer than 25 data points to violin plots, and also included the individual data points to better represent the distribution of the data from each sample.

- the authors suggest that xol-1 is not a transcriptional regulator of SDC proteins but does regulate SDC-2, perhaps at the level of mRNA translation. The authors could analyze SDC-2 foci by immunofluorescence and compare wild type and xol-1 null mutants to test if their null mutant regulate SDC-2 protein level.

We thank the reviewer for suggesting this insightful experiment. We have performed intensity quantification on SDC-2 foci at 50-100 cell stages, using HTZ-1 as an internal staining control in Supplementary Figure 2C of the revised manuscript. This quantification reveals that, as measured by IF, total SDC-2 protein intensity is lower in a *xol-1* mutant background compared to WT. In Supplementary Figure 3D, we also see that the mRNA levels of *sdc-2* are also decreased in *xol-1* mutants compared to WT. Together, these suggest that XOL-1 does not act at the level of mRNA transcription or translation. Rather, it is likely that XOL-1 inhibits in the activity of the SDC-2 protein through protein-protein interactions or some other post-translational mechanisms, which is why we see accelerated loading of the SDC-2 protein onto the X chromosomes in a *xol-1* mutant background. We are interested in pursuing these open questions surrounding the mechanistic bases of XOL-1 action in future studies.

Reviewer #2: This paper investigates the role of xol-1 in C. elegans hermaphrodites. The authors show that there is an acceleration of embryogenesis from the 8-cell stage to bean stage by 13 min in xol-1 mutants. This correlates with a large number of genes changed during early embryogenesis but these gene expression changes go away by the L1 stage. XOL-1 represses hermaphrodite sex determination genes, so there should be an up regulation of these genes in mutants. XOL-1 activates male sex determination so there should be an down regulation of these genes in mutants. Both of these are observed. However, this does not lead to any strong phenotypes. For example, there is no difference in viability at either stage. DPY-27 and SDC-2 load onto the X chromosome slightly earlier at the 50-100 cell stage in xol-1 mutants. There is also more nuclear accumulation of MET-2 in xol-1 mutants and this corresponds with a decrease in H3K9me2 and a later accumulation of H3K9me3, consistent with K9me2 being converted to K9me3 at a higher rate in xol-1 mutants. Consistent with this, the DCC loading acceleration in xol-1 mutants seems to be partially dependent upon met-2. Furthermore, in the met-2; xol-1 double the acceleration of embryogenesis is reverted, but this reversion is well past the met-2 single mutant (which is normal) suggesting there is a genetic interaction more than there is simply a dependence.

Main comments
The paper was very clearly written and easy to read. Overall, the results that the authors observe are quite subtle, indicating that xol-1 does not have a very large function in hermaphrodites. But, on the other hand, the results are internally consistent, suggesting they are likely to be real. In the end, I believe the results are interesting and novel enough to warrant publication. However, with the changes that the authors observe being quite subtle, my main concern is whether the numbers that were assayed were large enough.

The acceleration difference across 4.5hrs is only 13 min. I realize that this is statistically significant, but the N is only >6. I think this needs to be done on far greater numbers of embryos to make the result stronger.

To address this concern, we have increased the N for the time-lapse imaging assay. We have now scored a minimum of 11 embryos per genotype. For N2 and *xol-1* mutants, we have scored 15 and 16 embryos respectively. With this increase in the number of embryos scored, the difference in the transition time from the 8-cell stage to the bean stage remains comparable to what was reported in the first version of our study i.e. N2 takes 4h 43min and *xol-1 (ne4472)* mutants takes 4h 29min to complete this transition, representing a difference of ~14mins, and this difference remains statistically significant. We are unable to further increase the N numbers in the time given to us to complete revisions, as only 1-2 embryos can be imaged in one day over the course of 6-8 hours. However, given that the results remain the same after increasing the N number by more than two-fold, and the results are also consistent with the those from the rest of the study, we are confident that these numbers are sufficient to obtain measurements that represent real biological differences between the genotypes.

The gene expression changes are consistent with xol-1 embryos being developmentally accelerated also, but again the changes are really small. For the RNAseq in figures 1 and 4, there is no N indicated for number of replicates. With the small gene expression changes, the number of replicates should be at least 3 and perhaps 5.

The RNA-seq results from Figure 1 and 4 represent data from three replicates per genotype for all the datasets reported in our study. We have added the information about the number of replicates used for RNA-seq experiments to the Materials and Methods section of the revised manuscript.

There are no N for the number of embryos that were scored in figure 3.

In our revised manuscript, we have included details about the number of embryos quantified in the foci formation assays reported in Figure 3 in the figure legends. For Figure 3B, we quantified 82 and 69 embryos at 50-100 cell stages for N2 and *xol-1 (ne4472)* respectively. For Figure 3D, we quantified 73 and 62 embryos at 20-50 cell stages for N2 and *xol-1 (ne4472)* respectively. For Figure 3F, we quantified 32 and 48 embryos at 50-100 cell stages for N2 and *xol-1 (ne4472)* respectively. For Figure 3G, we quantified 14 and 16 embryos at 20-50 cell stages for N2 and *xol-1 (ne4472)* respectively.

In figure 5D,E there is no N for number of embryos

To address the next point below about internal staining control, the staining and quantification for Figure 5 were redone with internal staining controls to normalize the signal from our protein of interest. We quantified a minimum of 20 nuclei per embryo, representing nuclei from at least 7 different embryos at 20-50 cell stages for Figure 5D, and 10 different embryos at 50-100 cell stages for Figure 5E. We have added this information to the figure legends for Figure 5 in the revised manuscript.

In figures 3 and 5, there is no internal staining control for normalization of the signal. This should be added.

The foci formation assays shown in Figure 3 and Figure 7 included an internal staining control to ensure proper staining of embryos, and to make sure that the results from quantification can be compared between genotypes. We used antibodies against the nuclear pore complex as the internal staining control. We have now included this information in the figure legends, and added images of the staining controls to Figure 3, Figure 7 and Supplementary Figure 2.

For the experiment quantifying MET-2 protein levels in Figure 5, we repeated the immunofluorescence staining experiment with an internal staining control against the histone variant HTZ-1 to measure MET-2 intensity in a WT and *xol-1 (ne4472)* background. MET-2 protein levels appear to be significantly increased in *xol-1* mutants compared to WT at the 50-100 cell stages (Figure 5E). However, at the earlier 20-50 cell stage, MET-2 levels appear to be significantly lower in *xol-1* mutants compared to WT.

In figure 6, again no N for number of embryos

In Figure 6A-C, and 6E, we quantified 21 nuclei per genotype, representing nuclei from at least 7 embryos for every genotype. We have included this information in the figure legends in the revised edition of the paper.

In figure 7A, there is no WT, so it is not possible to know whether met-2 mutants are actually slower to load the DCC complex. This should be added.

We repeated the DPY-27 foci formation assay with WT and *met-2* mutant strains to determine whether *met-2* mutants are slower to load the DCC complex (Supplementary Figure 5). We quantified 48 and 52 embryos for N2 and *met-2* mutants respectively. Our results show that WT and *met-2* mutants have a similar rate of foci formation at the 50-100 cell stages, with the differences between the two being statistically non-significant using a chi-square test.

In figure 7 there is also no N number of embryos scored

In Figure 7A, we quantified >120 embryos per genotype. We have included this information in the figure legends in the revised version of the paper.

Minor Comments

What stage were the embryos for RNAseq? Perhaps I missed it but I think it just says early embryos.

The early embryo datasets represent a distribution of embryo stages that is quantified in Figure 2A. The “early embryo” samples were synchronized in time, by allowing synchronized L1 larvae to feed on a bacterial food source for 72hr. At this stage, the young adults were bleached to obtain embryos. More details about our procedure for the generation of RNA-seq datasets can be found in the Materials and Methods. We call these “early embryo datasets” because in the wild-type N2 samples, these time-synchronized embryos represent a distribution of embryo stages that peaks at the early 50-100 cell stage (Fig 2A).

How was fertilized vs. unfertilized determined?

Fertilized embryos and unfertilized oocytes differ significantly in their appearance, and can hence be distinguished easily using a stereo dissecting microscope. Geldziler et al. (2011) described this method to distinguish fertilized embryos and unfertilized oocytes using their appearance. Fertilized embryos appear oblong and are surrounded by an eggshell that is secreted by the embryo shortly after fertilization. In contrast, unfertilized oocytes appear circular in shape, and lack the eggshell characteristic of fertilized embryos.
